# Supplementary material for: HIV-1 diverts cortical actin for particle assembly and release
Source: Nat Commun. 2023 Oct 31;14:6945. doi: 10.1038/s41467-023-41940-0 (PMC10618566; doi:10.1038/s41467-023-41940-0)

SUPPLEMENTARY INFORMATION

HIV-1 diverts cortical actin for particle assembly and release.

Rayane Dibsy[1], Erwan Bremaud[1], Johnson Mak[2], Cyril Favard[1] and Delphine Muriaux[1]*.

Affiliations

[1]Institute of Research in Infectious disease of Montpellier (IRIM), University of Montpellier, UMR9004 CNRS, Montpellier, France

[2]Institute for Glycomics, Griffith University, Brisbane, Australia

[*] Correspondence to be addressed to Delphine Muriaux (delphine.muriaux@irim.cnrs.fr)

Supplementary Figure 1 :

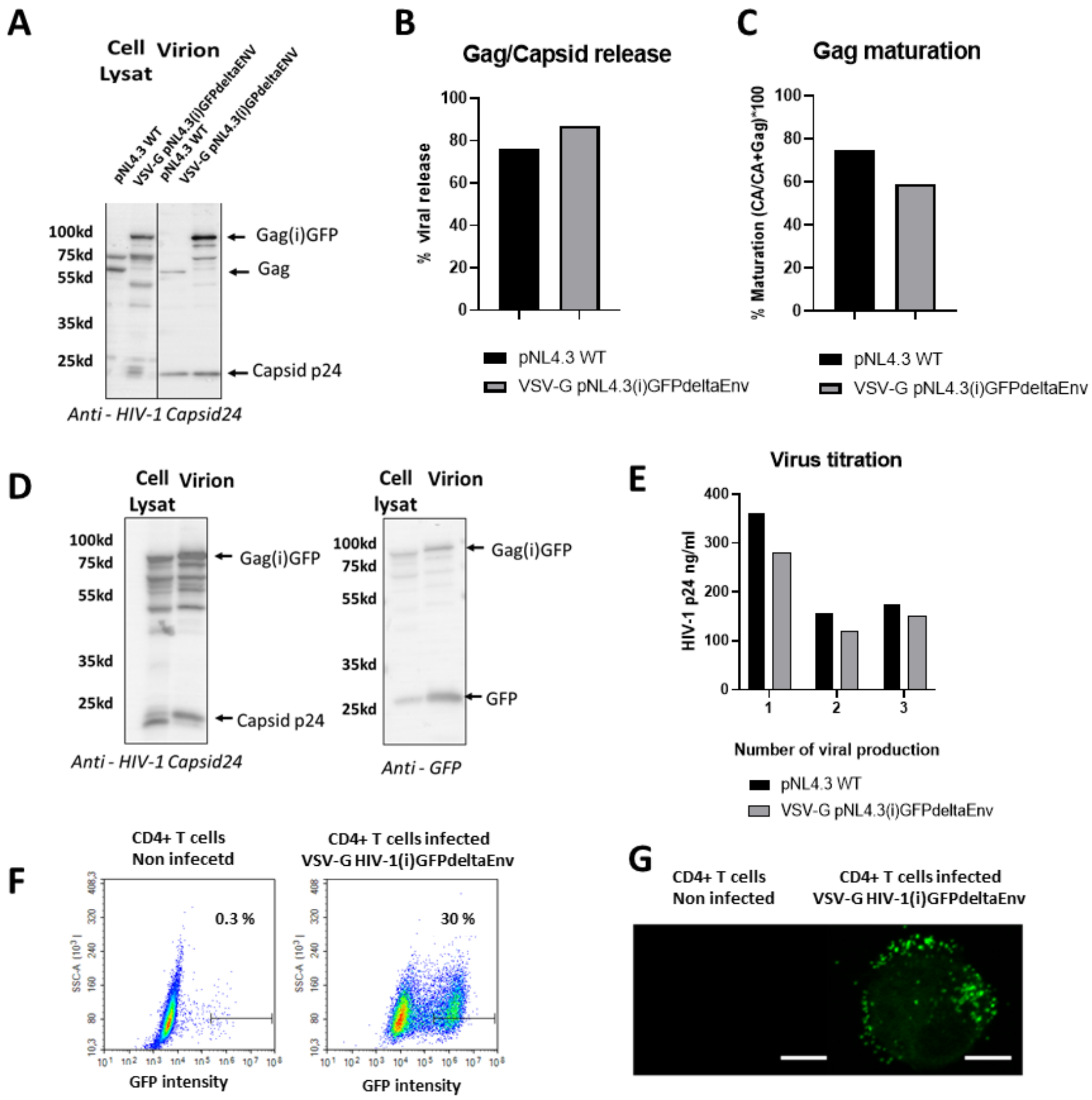

Supplementary Figure 1. Validation of pNL4.3(i)GFPdeltaEnv virus production and infectivity. *A) Immunoblot showing Gag pr55 as well as Capsid (CAp24) in cell lysate and in virion of 293T HEK cell line transfected with pNL4.3 Wild Type HIV-1. In addition, HIV-1 Gag(i)GFP is shown in cells transfected with pNL4.3(i)GFPdeltaEnv. B) Histogram showing the quantification of viral release from: cell transfected with pNL4.3 Wild type and pNL4.3(i)GFPdeltaEnv corresponding to the western blot in A. C) Histogram showing the quantification of Gag maturation in both condition. D) Immunoblot showing intracellular Gag(i)GFP expressed in cells transfected with pNL4.3(i)GFPdeltaEnv as well as extracellular Gag(i)GFP and its cleaved mature capsid p24 in the virion revealed by an anti-p24 Capsid antibody (left blot). Free GFP was revealed with an anti-GFP antibody (right blot). E) Histogram showing the titration of viruses produced and purified from 293T HEK cell line transfected with pNL4.3 WT or VSV-G pseudotyped pNL4.3(i)GFPdeltaEnv. Virus titration quantified by alphaLISA from three different productions #1, #2 and #3. Each production done on the same day allows a comparison of the 2 types of viruses. F) Flow cytometry showing the level of infectivity of VSVG-pseudotyped HIV-1(i)GFPdeltaEnv virus on CD4+ T Jurkat T cells (n=20 000 events). G) STED images of Jurkat T cells infected and non-infected with the VSV-G pseudotyped HIV-1(i)GFPdelta Env showing Gag(i)GFP in green. Scale bar is 5 µm.*

Supplementary Figure 2 :

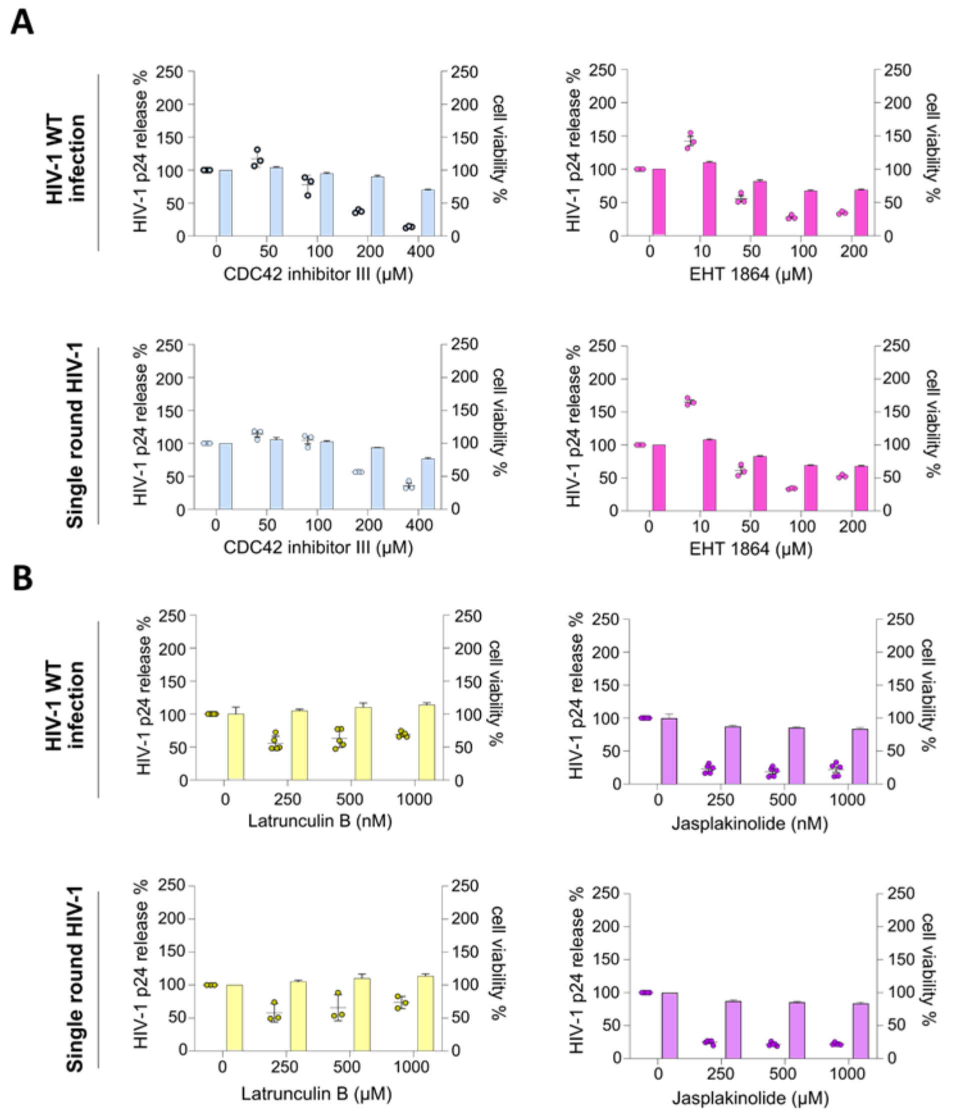

***Supplementary Figure 2 : Effect of actin interfering drugs on infected primary lymphocytes**. Left Y axis represents the relative percentage of HIV-1 p24 release (scatter plot) from infected activated primary blood lymphocytes (PBL) with HIV-1 Wild type (WT) in the upper panel, and VSVG-pseudotyped HIV-1(i)GFPΔEnv virus (single round infection virus) in the bottom panel, treated 24 hours post infection with A) 0, 50, 100, 200, and 400 µM of CDC42 III inhibitor (in blue), and 0, 10, 50, 100 and 200 µM of Rac1 inhibitor, EHT1864, (in pink), and B) 0, 0.25, 0.5 and 1 µM of LatrunculinB (in yellow) and 0, 0,25, 0,5 and 1 µM of Jasplakinolide (in purple). Percentage of viral release is normalized to the control (zero drug). Right Y axis represents the percentage of cell viability (bars). Percentage of cell viability is normalized to the control (zero drug). A) (N=1 donor, n=3 replicates). B) N=2 donors; n=5 replicates for HIV-1 WT infection and N=1 donor, n=3 replicates for single round infection. Data are presented as mean values +/- SD.*

Supplementary Figure 3 :

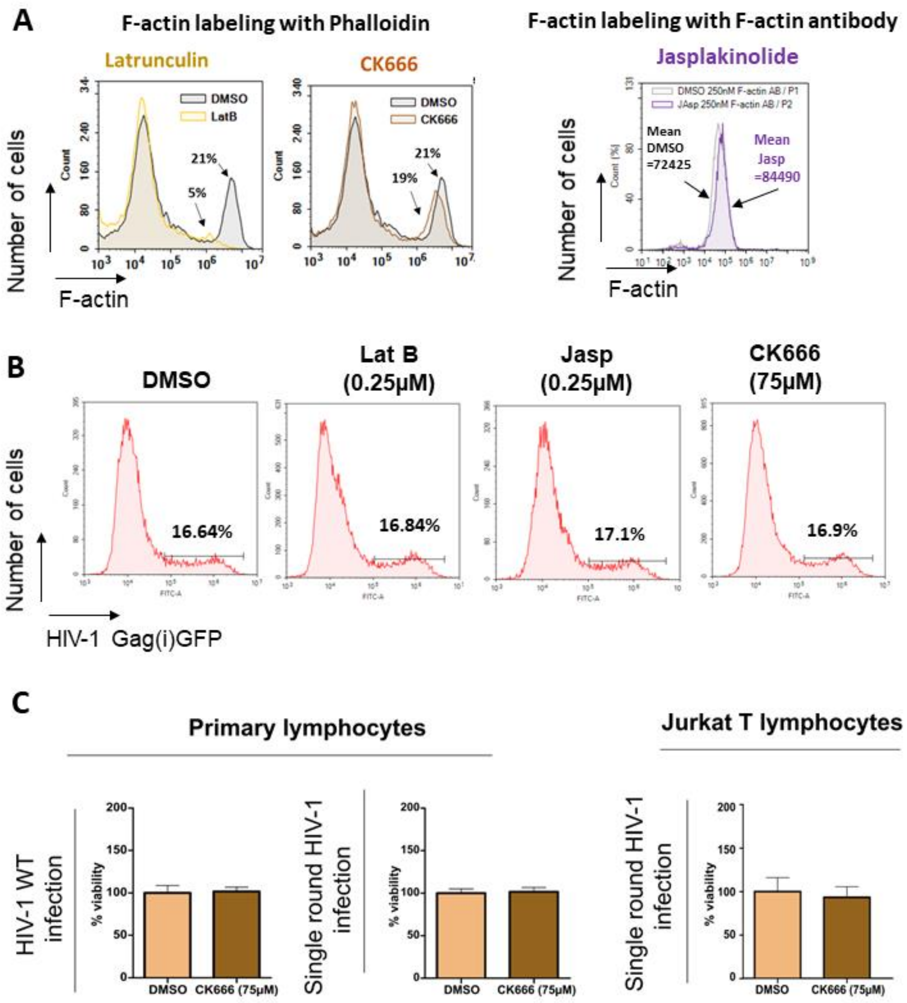

***Supplementary Figure 3** : **F-actin quantification post drug treatment.** A) Flow cytometry measuring intensity of Phalloidin-Alexa Fluor 647, on infected Jurkat T cells treated with LatrunculinB (yellow curve), or with CK666 (orange curve) in comparison to control DMSO (gray curve), or measuring intensity of Alexa fluor 647 anti-mouse antibody targeting F-actin for cell treated with Jasplakinolide (purple curve). B) Intracellular GFP quantification. Flow cytometry measuring intensity of GFP, on Jurkat T cells infected with VSVG-pseudotyped HIV-1(i)GFPΔEnv and treated with LatrunculinB, Jasplakinolide, CK666 or DMSO (control). N= 20 000 events. C) Percentage (%) of cell viability post treatment with 75µM of CK666 on infected primary blood lymphocytes (left) and CD4+ Jurkat T cell (right). Data are presented as mean values +/- SD.*

Supplementary Figure 4 :

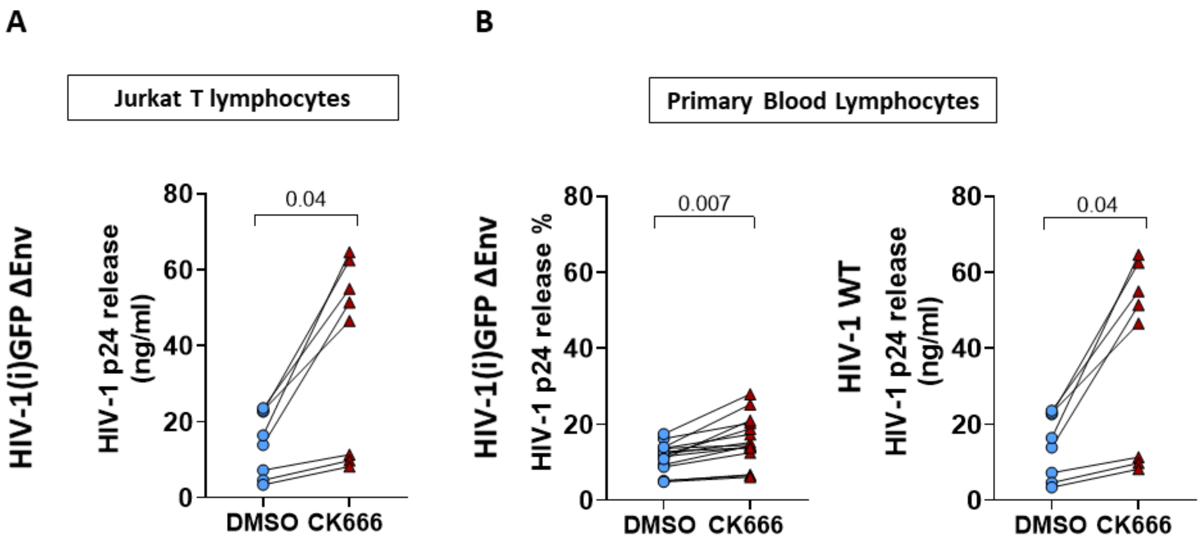

Supplementary Figure 4: Actin debranching favors HIV-1 release from infected CD4+ T lymphocytes. *Graphs showing the raw values of HIV-1 p24 release quantified by alphaLISA (HIV-1 p24 ng/ml) in supernatant of cells treated (red triangles) or not (blue dots) with CK666. Values coming from individual experiments are linked for clarity.*

Supplementary Figure 5 :

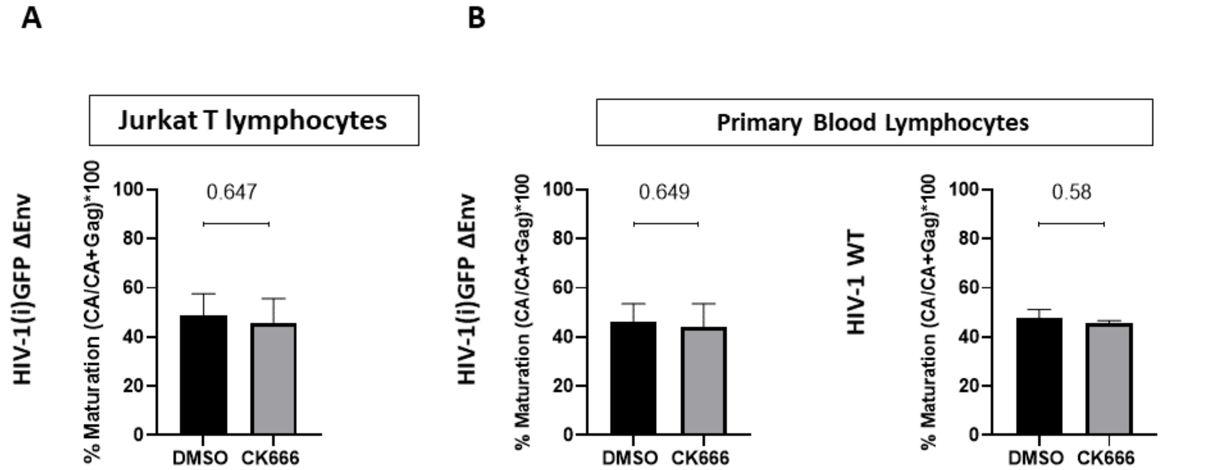

Supplementary Figure 5 : CK666 treatment has no effect on Gag maturation*. Histograms showing the percentage of Gag maturation calculated from western blot showed in Figure 2. % of Gag maturation is equal to Capsid/(Total=Capsid + Gag)*100, in infected Jurkat T cells in (A) (for N=6 independent experiments), and in infected primary blood T cells in (B) for single round infection (for N=7 independent experiments, 2 donors) or with WT HIV-1 (for N=10 independent experiments, 2 donors). Data are presented as mean values +/- SD. Exact p-values of a two-tailed Mann-Whitney test are given in graphs.*

Supplementary Figure 6 :

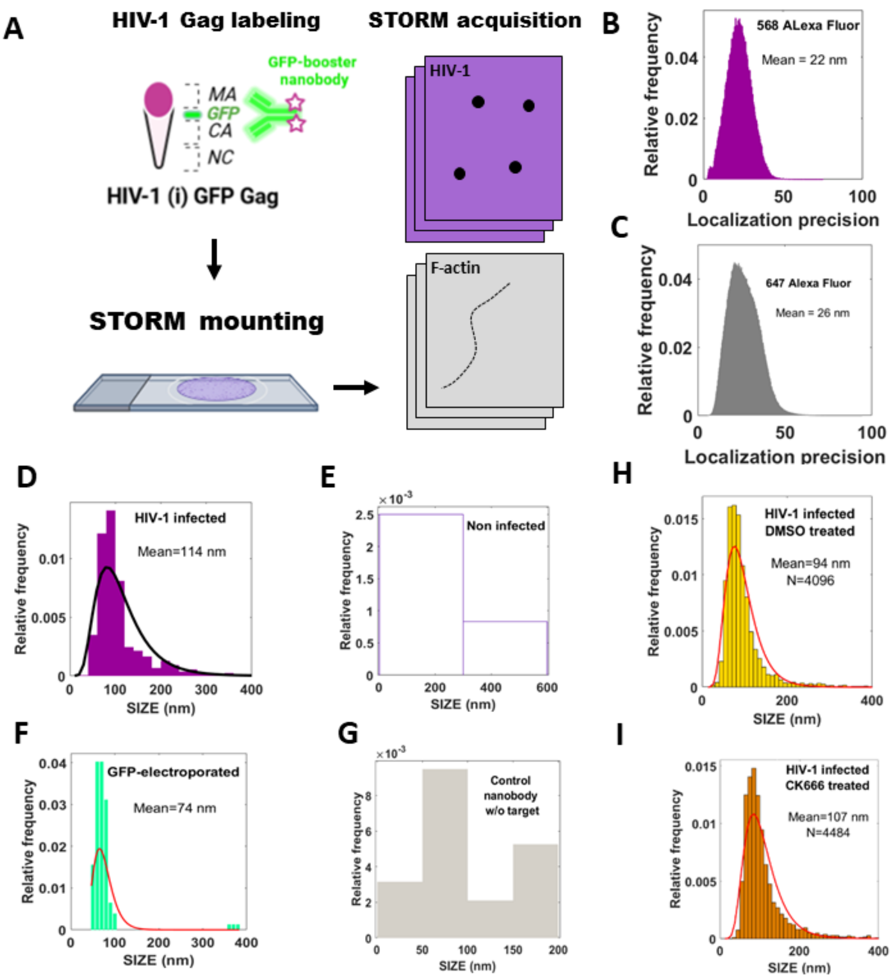

***Supplementary Figure 6** : **Protocol principle used to image HIV-1 and F-actin using STORM.** A) Scheme describing the protocol used to label HIV-1 Gag(i)GFP and F-actin for STORM imaging. B) and C) show the plot of localization precision of respectively Alexa fluor 568 (in purple) and Alexa fluor 647 (in gray). Controls used for nanobody-GFP specificity and clustering analysis. Histogram showing size distribution of HIV-1 Gag assembly clusters at the plasma membrane of infected CD4+ T lymphocytes in D), of unspecific clusters at the plasma membrane of non-infected CD4+ T lymphocytes in E), of GFP cluster in cytoplasm of pEGFP electroporated CD4+ T lymphocytes in F), and of nanobody-GFP alone sticking on the coverslip in G). H) histogram showing size distribution of HIV-1 Gag assembly clusters at the plasma membrane of infected CD4+ T lymphocytes treated with DMSO and in I) treated with CK666.*

Supplementary Figure 7 :

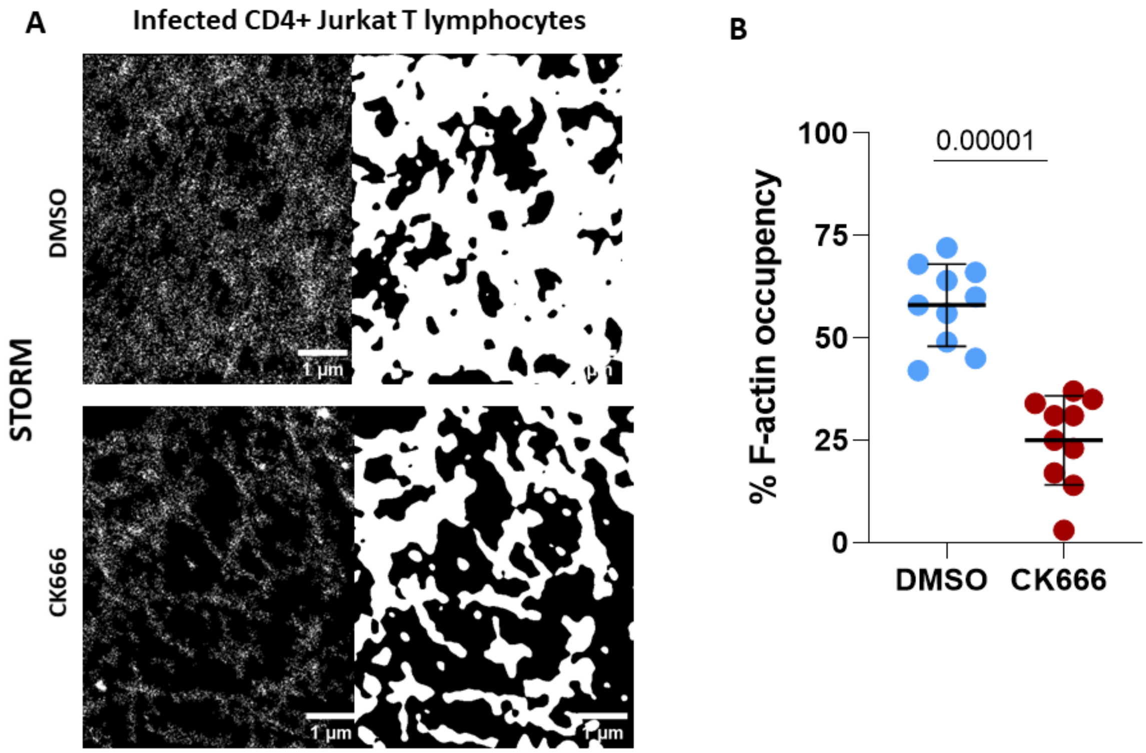

Supplementary Figure 7: STORM imaging of F-actin in infected Jurkat T cells. *A) Region-of-interest (ROI) of infected CD4+ Jurkat T lymphocytes showing actin filament stained with Phalloidin Alexa Fluor 647 and treated with DMSO (upper panel) or with 75µM CK666 (bottom panel). In the left, original STORM images are shown, and in the right their correspondent binary images. Scale bar is 1µm. B) dot plot showing the percentage of F-actin occupancy calculated from binary images in A). N=10 ROI. DMSO in blue, CK666 in red. Data are presented as mean values +/- SD. Exact p-values of a two-tailed Mann-Whitney test are given in graphs.*

Supplementary Figure 8 :

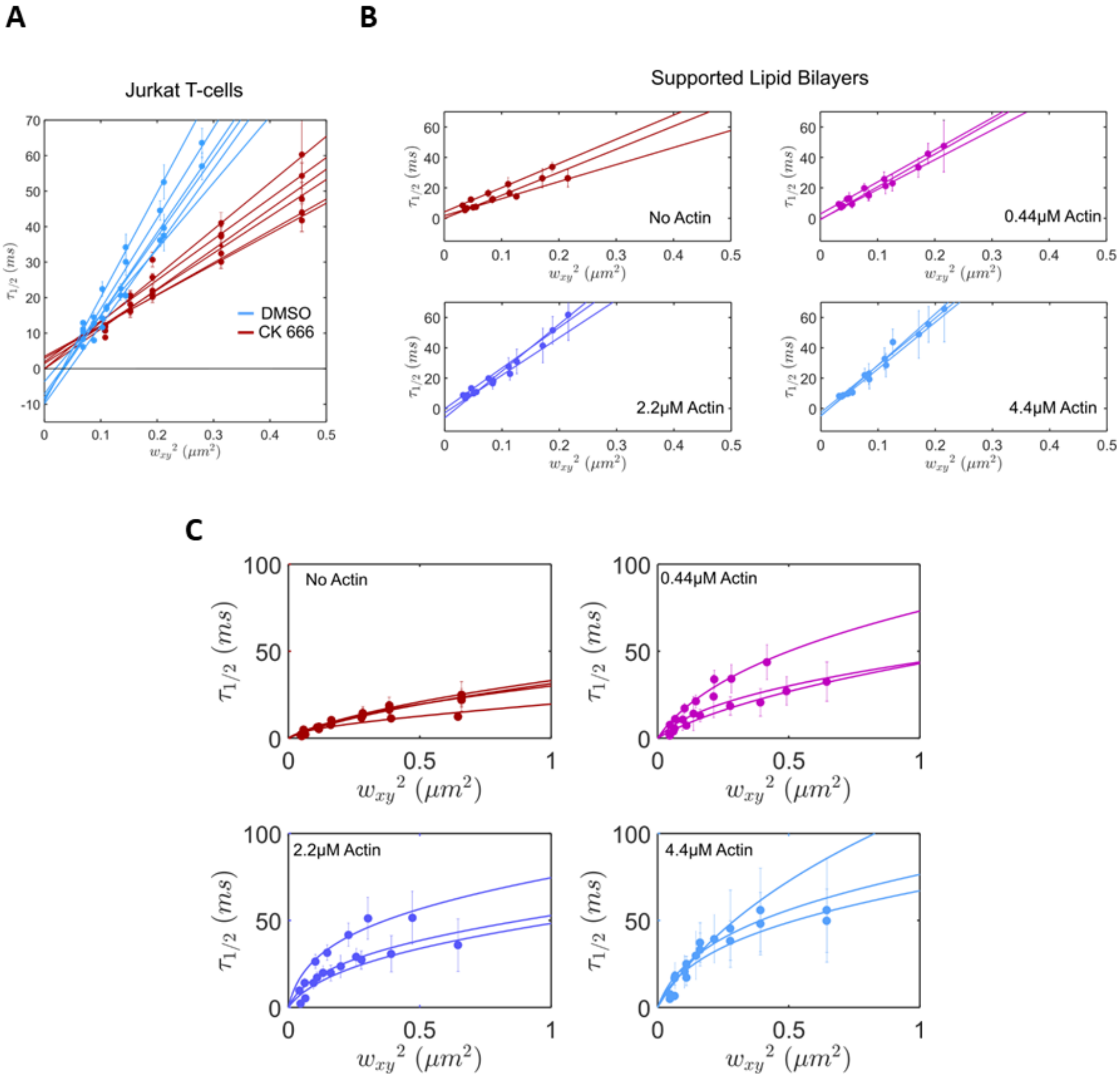

Supplementary Figure 8: Spot variation FCS diffusion laws in Jurkat T cells and in SLBs in different conditions. *A) Atto647N-PI(4,5)P2 lipid analogue spot variation FCS diffusion laws obtained in Jurkat T cells treated (red lines and dots) or not (blue lines and dot) with CK666. Lines are the best linear fits of the different half decorrelation times ($\tau_{1/2}$) measured at different laser waist (w^2_{xy}). N= 3 independent experiments, n= 6 cells for DMSO and for CK666. Each dot represents the mean value and the standard error of the mean of the decorrelation times ($\tau_{1/2}$) at one waist obtained from 20<n<50 different correlograms. B) Atto647N-PI(4,5)P2 lipid analogue spot variation FCS diffusion laws obtained in different (N=3) SLBs of the same lipid composition, with increasing actin meshwork density (from no Actin, to 4.4 µM Actin, see main text for detailed explanation). Each dot represents the mean value and the standard error of the mean of the decorrelation times ($\tau_{1/2}$) at one waist obtained from 20<n<50 different correlograms. C) Atto488-Gag spot variation FCS diffusion laws obtained in different (N=3) SLBs of the same lipid composition, with increasing actin meshwork density, as for Atto647N-PI(4,5)P2. Line are the best fit obtained with equations 6 and 7 of* [42]*. Each dot represents the mean value and the standard error of the mean of the decorrelation times ($\tau_{1/2}$) at one waist obtained from 20<n<50 different correlograms. Different colors represent different actin concentration.*

Supplementary Figure 9 :

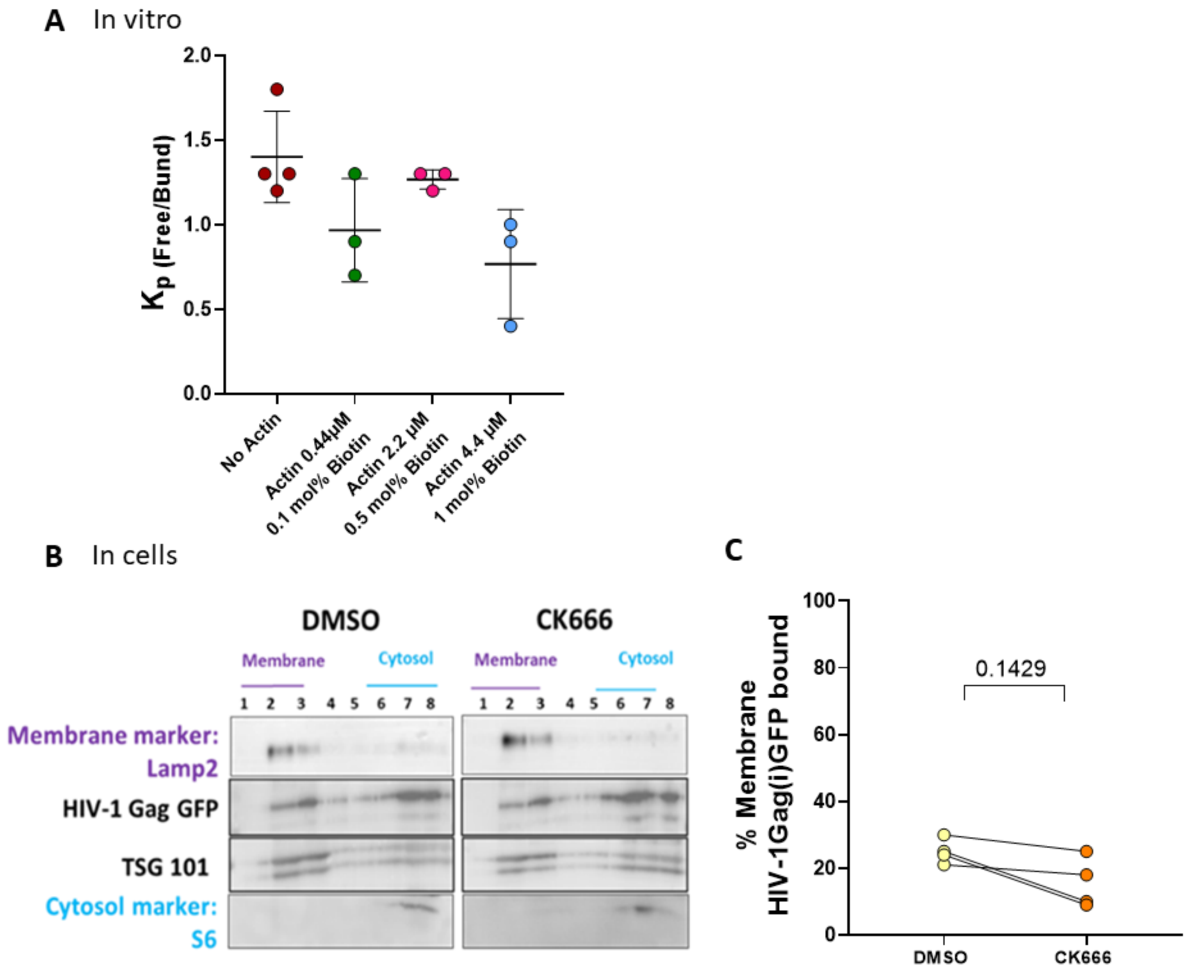

Supplementary Figure 9: Actin debranching does not affect HIV-1 Gag membrane binding. *A) Constant of partition of HIV-1 Gag on SLB with different actin concentrations. Different colors represent different actin concentrations. 3≤N≤4 different supported lipid bilayers with the same lipid composition were tested. B) Immunoblot showing HIV-1 Gag(i)GFP as well as TSG101 bands in membrane fractions (1, 2 and 3) and in fractions corresponding to the cytosol (6, 7 and 8) in presence or absence of CK666. Lamp2 was used as a membrane marker and ribosomal S6 as a cytosol marker. C) Dot plot showing the percentage (%) of cell membrane binding of HIV-1 Gag in infected Jurkat T cells treated or not with CK666 (N=4 independent experiments). Exact p-value of a two-tailed Mann-Whitney test is given in the graph.*

Supplementary Figure 10:

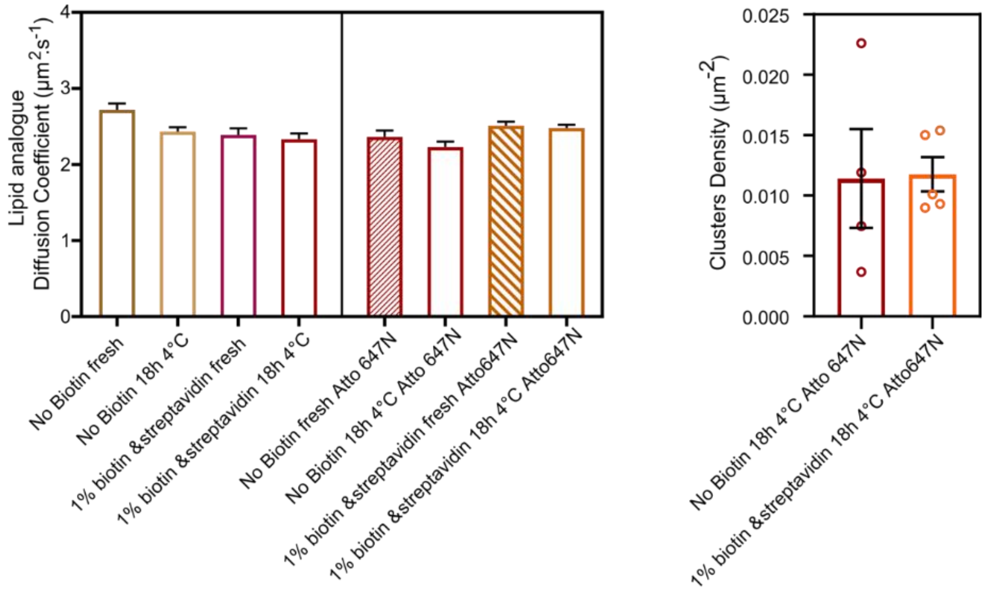

Supplementary Figure 10: Lipid mobility and Gag clusters on SLBs in different conditions in vitro. *Left: Lipid mobility and supported lipid bilayer (SLB) fluidity tested after 18h at 4°C. Both Cy5.5 DPPE (Bar graphs 1,2,3,4, left) and Atto647N-PI(4,5)P2 (Bar graphs 5,6,7,8, right) were used to test the membrane fluidity and integrity. 5 to 10 different zones of the SLB were tested and at least 20 correlograms for each zone were acquired to determine the average diffusion coefficients of the lipid analogues. These diffusion coefficients are equivalent ($2.4 \leq D \leq 2.7 \mu m^2.s^{-1}$), independently of a 18h stay at 4°C for the SLB, and independently of the presence or not of the highest biotinylated lipid concentration (1% mol) and streptavidin and phalloidin-biotin, without actin. These diffusion coefficients are typical of fluid SLBs. Right: Effect of biotinylated lipid anchor on HIV-1 self-assembly on SLBs. To discard possible effect due to the combination of biotinylated lipids incubated with streptavidin and biotinylated phalloidin, in the absence of actin, we performed HIV-1 Gag self-assembly experiments and quantified the Atto647N-PI(4,5)P2 induced clusters, as in Fig. 5 H & I. Bar graphs exhibit no difference between SLBs without biotinylated lipids (N=4 different SLB, n=1 imaged area per SLB) and SLB with biotinylated lipids (N=2, 2<n<3 imaged area per SLB, error bars are s.e.m). Different colors represent different experimental conditions as indicated.*

Supplementary Video S1: *In vitro* PI(4,5)P2 cluster formation after Gag injection on SLBs labeled with PIP2-Atto647 (in red).

Supplementary Video S2: *In vitro* HIV-1 Gag cluster formation on SLBs with Alexa488-labeled Gag proteins (in green).

Supplementary Video S3: *In vitro* PI(4,5)P2 and HIV-1 Gag cluster formation after Gag injection on SLBs labeled with PIP2-Atto647 (in red) and with Alexa488-labeled myr(-)Gag proteins (in green). Merge PIP2 and Gag clusters appear in yellow color.

Source data file – Western Blots - Supplementary Figure 1.A

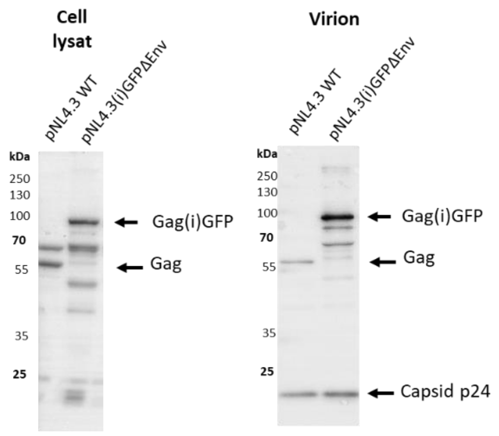

Source data file – Western Blots - Supplementary Figure 1.D

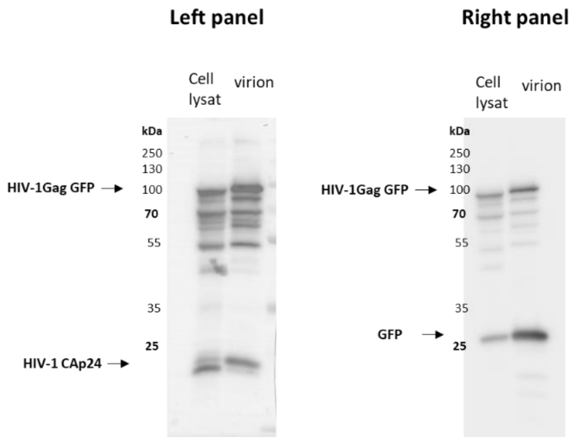

Source data file – Western Blots - Supplementary Figure 9.B

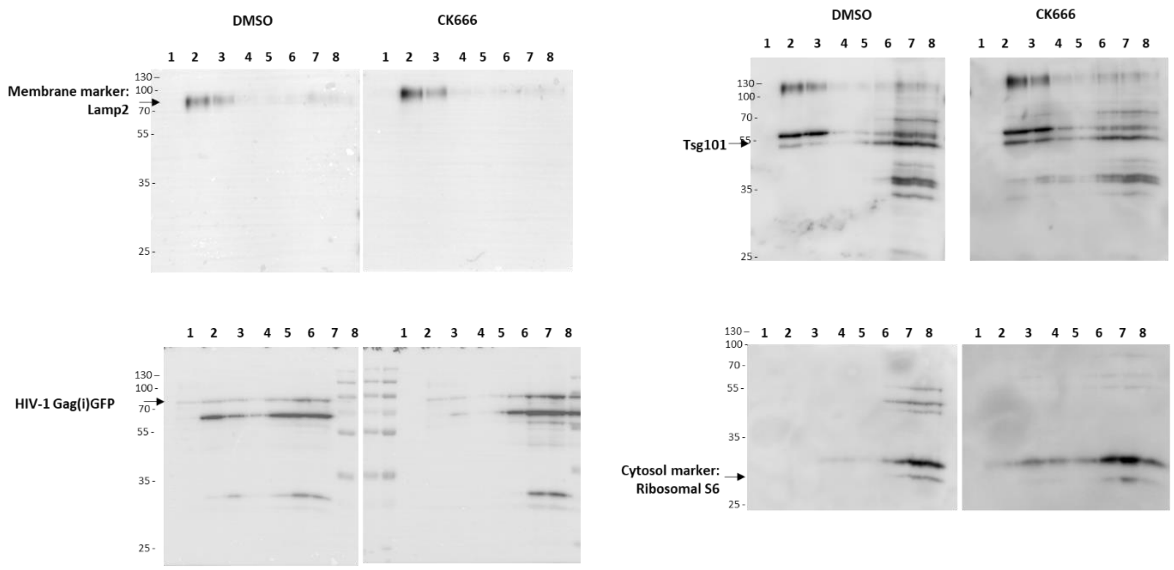

Supplement: Supplementary file 1 — Supplementary Information [file 41467_2023_41940_MOESM1_ESM.pdf]
