# Supplementary material for: HIV-1 diverts cortical actin for particle assembly and release
Source: Nat Commun. 2023 Oct 31;14:6945. doi: 10.1038/s41467-023-41940-0 (PMC10618566; doi:10.1038/s41467-023-41940-0)
Supplement: Supplementary file 3 — Description of Additional Supplementary Files [file 41467_2023_41940_MOESM3_ESM.pdf]

Description of Additional Supplementary Files

File Name: Supplementary Video S1
Description: *In vitro* PI(4,5)P2 cluster formation after Gag injection on SLBs labeled with PIP2-Atto647 (in red).

File Name: Supplementary Video S2
Description: *In vitro* HIV-1 Gag cluster formation on SLBs with Alexa488-labeled Gag proteins (in green).

File Name: Supplementary Video S3
Description: *In vitro* PI(4,5)P2 and HIV-1 Gag cluster formation after Gag injection on SLBs labeled with PIP2-Atto647 (in red) and with Alexa488-labeled myr(-)Gag proteins (in green). Merge PIP2 and Gag clusters appear in yellow color.
