# Supplementary material for: HIV-1 diverts cortical actin for particle assembly and release
Source: Nat Commun. 2023 Oct 31;14:6945. doi: 10.1038/s41467-023-41940-0 (PMC10618566; doi:10.1038/s41467-023-41940-0)
Supplement: Supplementary file 7 — Reporting Summary [file 41467_2023_41940_MOESM7_ESM.pdf]

nature portfolio

Corresponding author(s): Delphine MURIAUX
NCOMMS-22-50340A

Last updated by author(s): Sep 22, 2023

Reporting Summary

Nature Portfolio wishes to improve the reproducibility of the work that we publish. This form provides structure for consistency and transparency in reporting. For further information on Nature Portfolio policies, see our Editorial Policies and the Editorial Policy Checklist.

Statistics

For all statistical analyses, confirm that the following items are present in the figure legend, table legend, main text, or Methods section.

n/a	Confirmed	
☐	☒	The exact sample size (*n*) for each experimental group/condition, given as a discrete number and unit of measurement
☐	☒	A statement on whether measurements were taken from distinct samples or whether the same sample was measured repeatedly
☐	☒	The statistical test(s) used AND whether they are one- or two-sided<br>*Only common tests should be described solely by name; describe more complex techniques in the Methods section.*
☒	☐	A description of all covariates tested
☒	☐	A description of any assumptions or corrections, such as tests of normality and adjustment for multiple comparisons
☐	☒	A full description of the statistical parameters including central tendency (e.g. means) or other basic estimates (e.g. regression coefficient) AND variation (e.g. standard deviation) or associated estimates of uncertainty (e.g. confidence intervals)
☐	☒	For null hypothesis testing, the test statistic (e.g. *F*, *t*, *r*) with confidence intervals, effect sizes, degrees of freedom and *P* value noted<br>*Give P values as exact values whenever suitable.*
☒	☐	For Bayesian analysis, information on the choice of priors and Markov chain Monte Carlo settings
☒	☐	For hierarchical and complex designs, identification of the appropriate level for tests and full reporting of outcomes
☒	☐	Estimates of effect sizes (e.g. Cohen's *d*, Pearson's *r*), indicating how they were calculated

Our web collection on statistics for biologists contains articles on many of the points above.

Software and code

Policy information about availability of computer code

Data collection	Fluorescence Correlation Spectroscopy data were acquired using LSM780 Confocal microscopes with ZEISS Zen black software ZEN 2012 SP1 Black Edition - Version 8.1.3.484.
Data analysis	FCS correlograms were analyzed with PyCorrFit 1.1.7 software (Müller, P., Schwille, P. & Weidemann, T. PyCorrFit—generic data evaluation for fluorescence correlation spectroscopy. Bioinformatics 30, 25)<br>STORM data were extracted thanks to the ThunderStorm Plugin of Fiji imaging software (ImageJ v1.53t).<br>Spot variation FCS were analyzed with a home made developped script running under Matlab 2016b : https://gitlab.inria.fr/hberry/gag_svfcs<br>Mann Whitney statistical testing was performed with PRISM 8.3.0 software (graphPad).

For manuscripts utilizing custom algorithms or software that are central to the research but not yet described in published literature, software must be made available to editors and reviewers. We strongly encourage code deposition in a community repository (e.g. GitHub). See the Nature Portfolio guidelines for submitting code & software for further information.

Data

Policy information about availability of data

All manuscripts must include a data availability statement. This statement should provide the following information, where applicable:

- Accession codes, unique identifiers, or web links for publicly available datasets
- A description of any restrictions on data availability
- For clinical datasets or third party data, please ensure that the statement adheres to our policy

All relevant data supporting the key findings of this study are available within the article and its Supplementary Information files. Source data are provided as a Source Data file in a ZIP folder containing the data of each graph and in Supplemental information for the uncropped Western blots. Due to size constrains, relevant raw data of FCS and single molecule localisation microscopy (STORM) are provided and available at the following address 10.5281/zenodo.8366334, the remaining raw data are available upon reasonable request.

Research involving human participants, their data, or biological material

Policy information about studies with human participants or human data. See also policy information about sex, gender (identity/presentation), and sexual orientation and race, ethnicity and racism.

Reporting on sex and gender	PBLs are from healthy donor (sex F) from Etablissement du Sang Français (EFS) Montpellier France (Donors #72201546476 and # 72202498058). Informed consent was obtained for blood donation under the ethical regulations of the Etablissement du Sang Français (EFS) in Occitanie (OCPM), France.
Reporting on race, ethnicity, or other socially relevant groupings	n/a
Population characteristics	n/a
Recruitment	n/a
Ethics oversight	Our research complies with all relevant ethical regulations in agreement with Etablissement du Sang Francais (EFS OCPM), Montpellier, France regulations for laboratory research use only under the convention #21PLER2019-0106/ CNRS reference #208847. Informed consent was obtained for blood donation. The EFS Occitanie Laboratory obtained its accreditation NF EN ISO 15189 in date of 15 August 2014 (accreditation # 8-1636 - available on www.cofrac.fr). The Directory board of EFS Occitanie is Directeur : Dr Laurent Bardiaux; Directrice adjointe : Aude Thiery; Secrétaire général : Philippe Guignon. Dr Aurélie Conte, Biologiste responsable - aurelie.conte@efs.sante.fr

Note that full information on the approval of the study protocol must also be provided in the manuscript.

Field-specific reporting

Please select the one below that is the best fit for your research. If you are not sure, read the appropriate sections before making your selection.

☒ Life sciences ☐ Behavioural & social sciences ☐ Ecological, evolutionary & environmental sciences

For a reference copy of the document with all sections, see nature.com/documents/nr-reporting-summary-flat.pdf

Life sciences study design

All studies must disclose on these points even when the disclosure is negative.

Sample size	We do not predetermined our samples size, our samples size are in the standard of the already published scientific litterature in the field. Experiments were replicated to ensure statistical validity.
Data exclusions	For FCS, correlograms were discarded when the fluorescence trace (intensity fluctuation over time) was subjected to photobleaching. Photobleaching was monitored by changes in the average fluorescence value at long time compared to the average fluorescence value at the initial time of the recording. For biological samples gene extinction, data were discarded when the targeted gene was not knockdown by the corresponding siRNA or if the cells presented a high degree of mortality.
Replication	Each experiment was perfomed in 2 (for blood donors) to 10 independent biological replicates or model systems. All attempts at replication were successful.
Randomization	Randomization is not necessary or applicable to our study.
Blinding	No blinding was performed in this study since our experiments did not require this.

Reporting for specific materials, systems and methods

We require information from authors about some types of materials, experimental systems and methods used in many studies. Here, indicate whether each material, system or method listed is relevant to your study. If you are not sure if a list item applies to your research, read the appropriate section before selecting a response.

Materials & experimental systems

n/a	Involved in the study
☐	☒ Antibodies
☐	☒ Eukaryotic cell lines
☒	☐ Palaeontology and archaeology
☒	☐ Animals and other organisms
☒	☐ Clinical data
☒	☐ Dual use research of concern
☒	☐ Plants

Methods

n/a	Involved in the study
☒	☐ ChIP-seq
☐	☒ Flow cytometry
☒	☐ MRI-based neuroimaging

Antibodies

Antibodies used	The antibodies used in this study are:<br>- Mouse anti-CAp24 (6521 NIH aids reagent program),<br>- Rabbit polyclonal anti-Arpin (Sigma ABT251)<br>- Rabbit polyclonal anti-Arpin (Invitrogen PA5-98574)<br>- Anti-GAPDH HRP (Sigma G9295).<br>- GFP-Booster Alexa Fluor 568 nanobody (Chromotek gb2AF568).<br>- Anti-TSG101 antibody [EPR7130(B)] (Abcam ab125011).
Validation	All antibodies were validated by the manufacturers indicated above and available on the manufacturers websites.

Eukaryotic cell lines

Policy information about cell lines and Sex and Gender in Research

Cell line source(s)	Human Jurkat T lymphocytes (human T-cell leukemia cell line) (ATCC-CRL-2899TM)<br>Human embryonic kidney cells (293THEK cell) (HEK 293T-ATCC-CRL-1575TM)
Authentication	Authentification of cell lines were done by ATCC (The Global Bioresource Center | ATCC).
Mycoplasma contamination	All the cell lines tested negative for mycoplasms (test MycoAlert detection kit #LT07-318 from Lonza, done every month at the IRIM institute)
Commonly misidentified lines<br>(See ICLAC register)	No commonly misidentified cell line were used in this study.

Flow Cytometry

Plots

Confirm that:

☒ The axis labels state the marker and fluorochrome used (e.g. CD4-FITC).

☒ The axis scales are clearly visible. Include numbers along axes only for bottom left plot of group (a 'group' is an analysis of identical markers).

☐ All plots are contour plots with outliers or pseudocolor plots.

☐ A numerical value for number of cells or percentage (with statistics) is provided.

Methodology

Sample preparation	F-actin and intracellular Gag-GFP measurement were performed by flow cytometry. Briefly infected T cells expressing Gag-GFP were fixed with 4% PFA in PBS, and stained with phalloidin Alexa Fluor 647 or F-actin antibody with secondary conjugated to Alexa Fluor 647. After staining, samples are washed and resuspend with PBS. 20 000 events were analysed by Novocyte/FlowJo.
Instrument	Novocyte

Software	FlowJo
Cell population abundance	20000 events
Gating strategy	Gating was done on the main cell population regarding cell morphology (size and granulation).

☐ Tick this box to confirm that a figure exemplifying the gating strategy is provided in the Supplementary Information.
