# Supplementary figures and images for: HIV-1 diverts cortical actin for particle assembly and release
Source: Nat Commun. 2023 Oct 31;14:6945. doi: 10.1038/s41467-023-41940-0 (PMC10618566; doi:10.1038/s41467-023-41940-0)

Figure 1.C

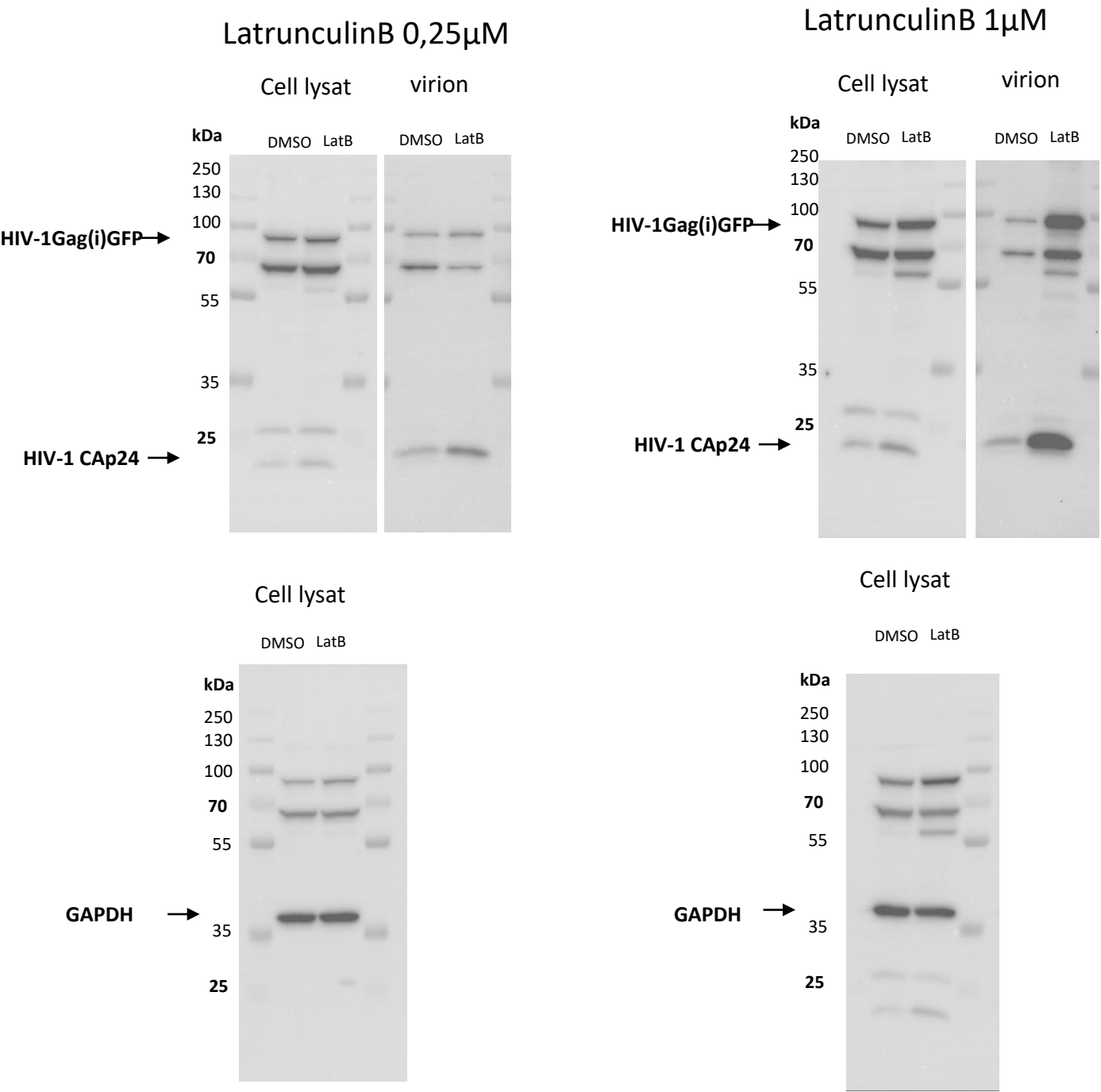

Figure 2.F

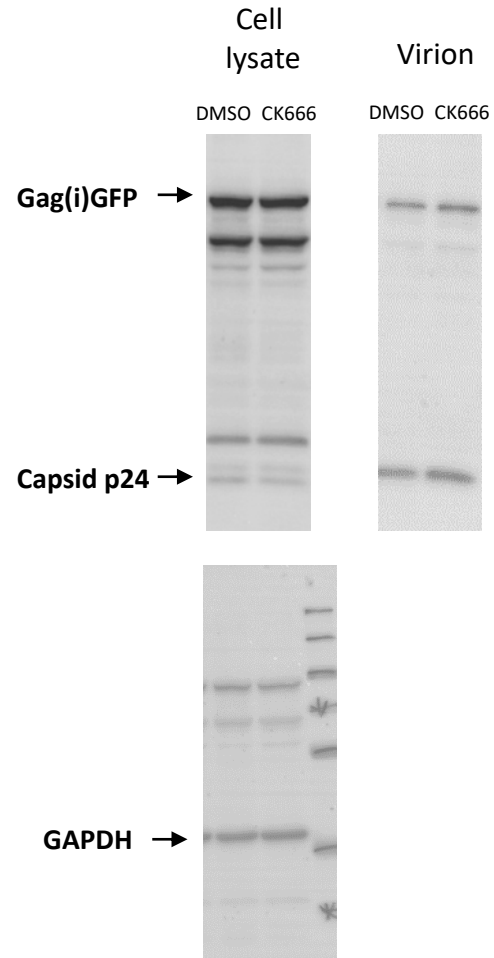

Figure 2.G (left panel)

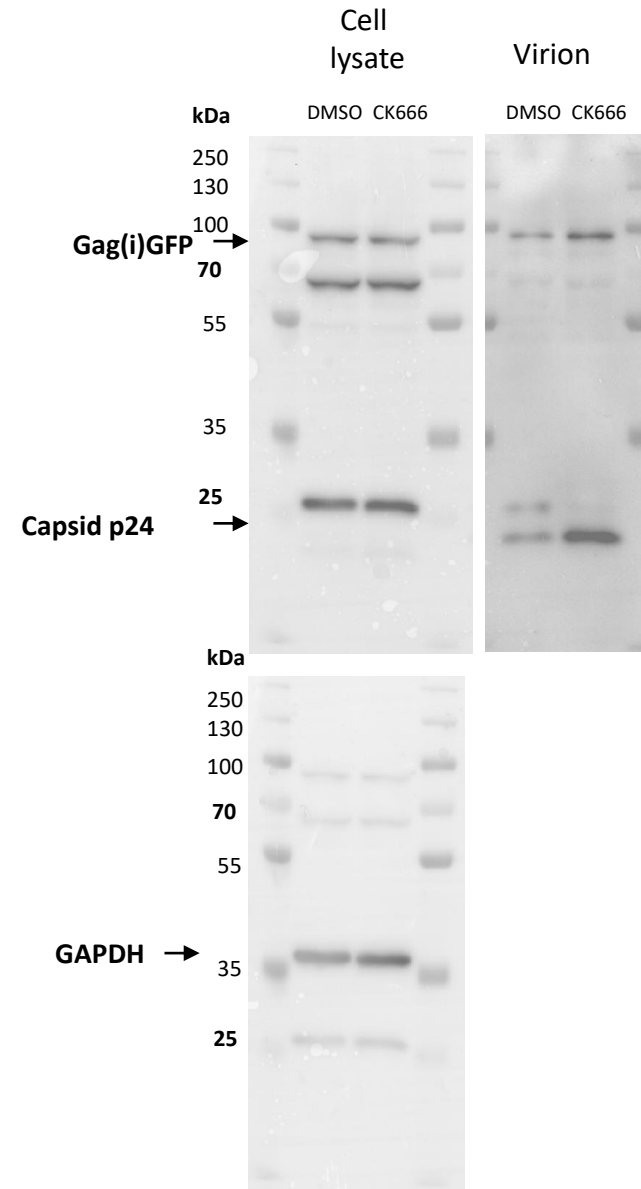

Figure 2.G (right panel)

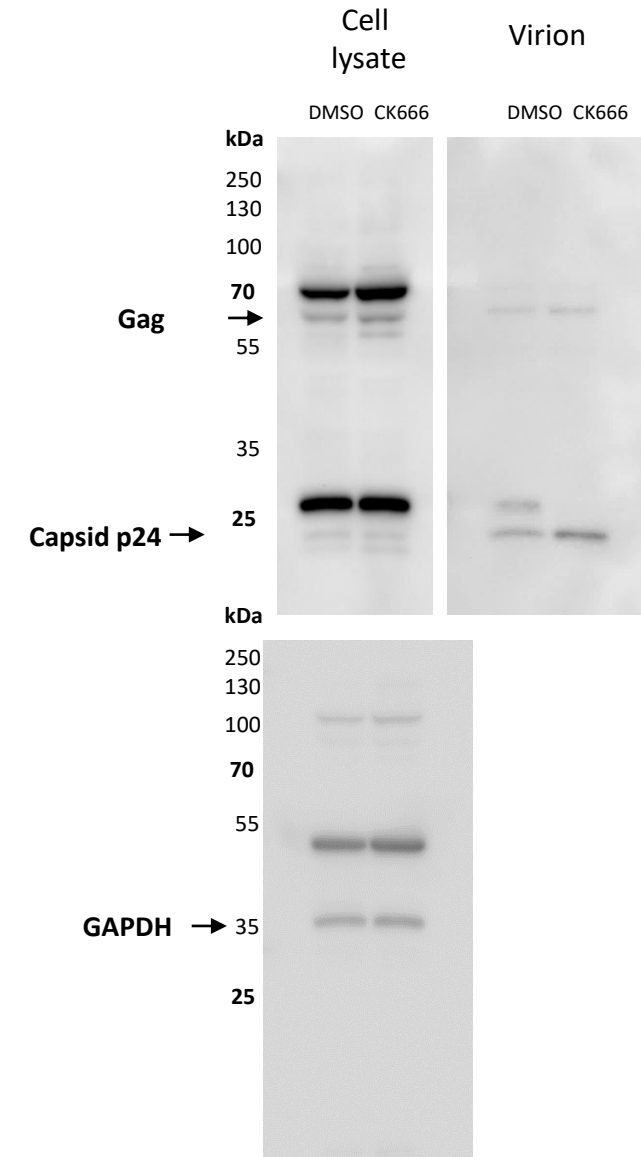

Figure 6.A

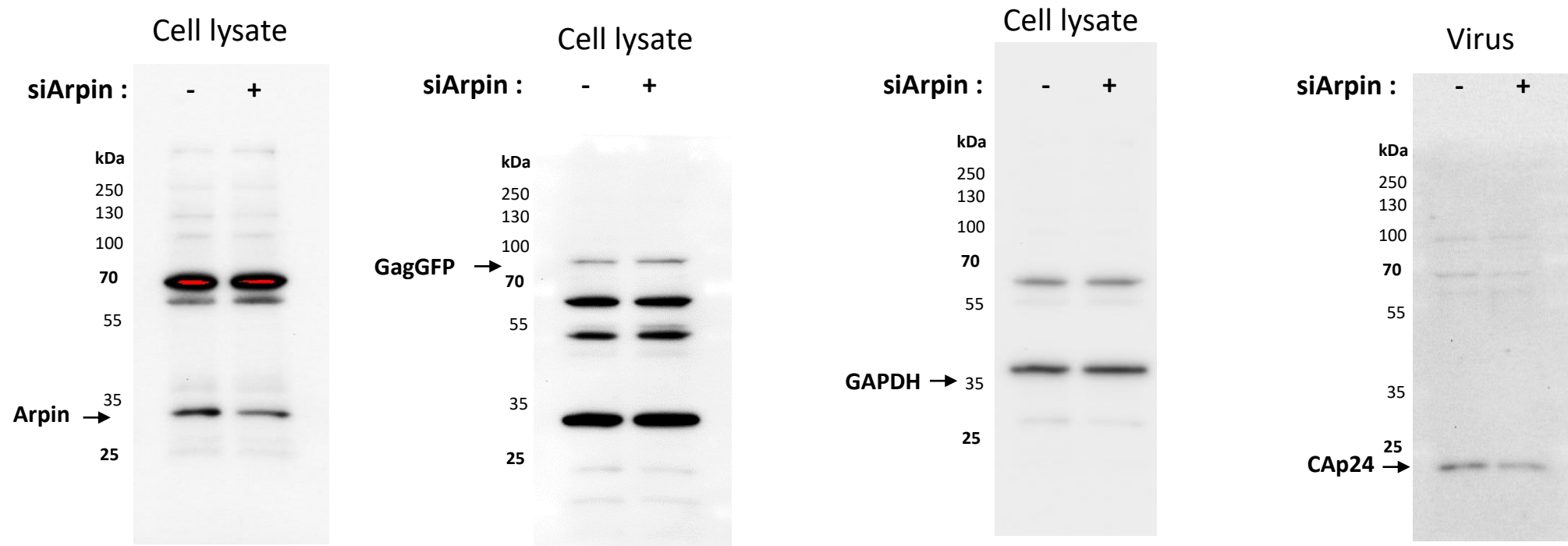

**Figure 7.B**

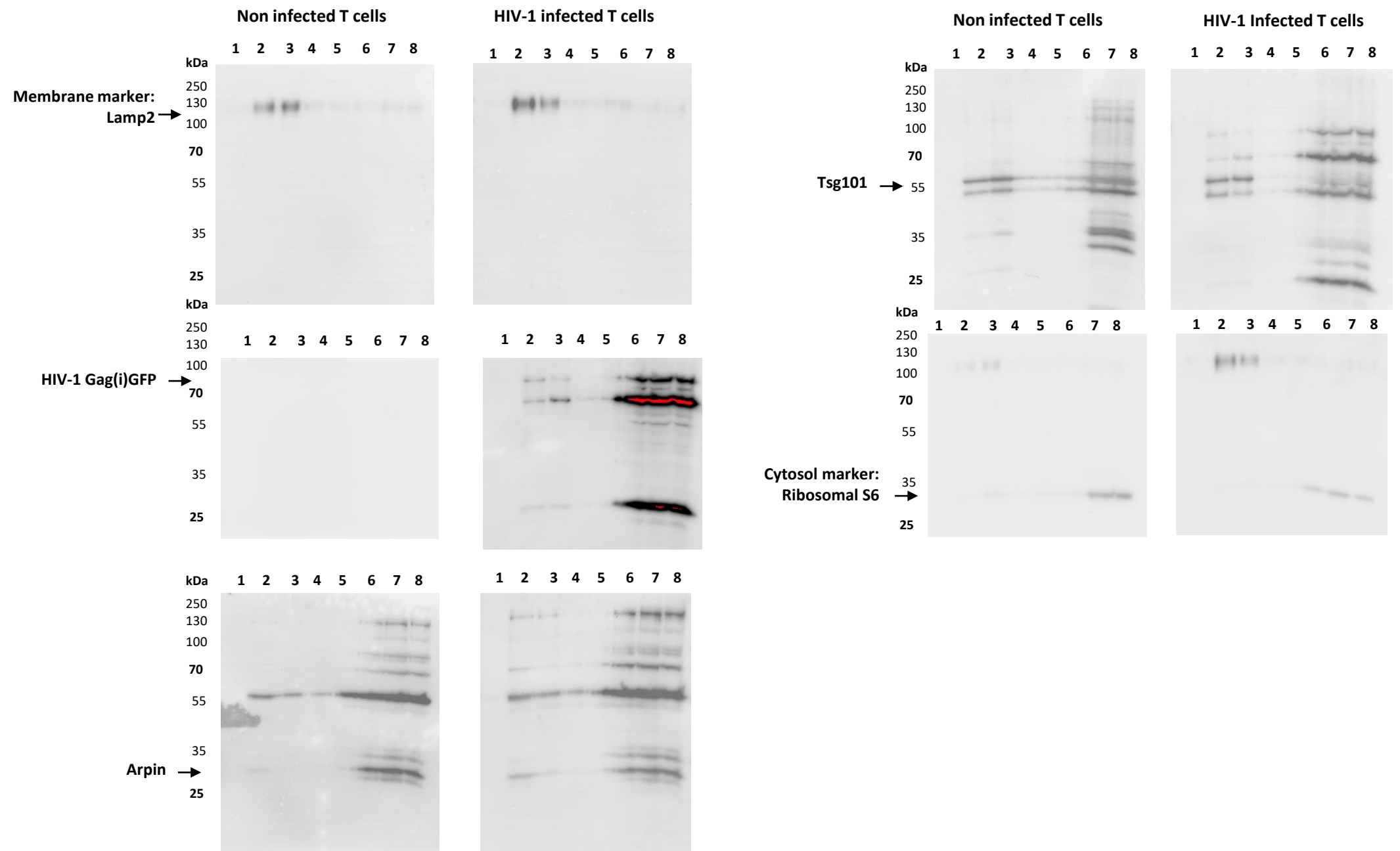

**Figure 7.E**

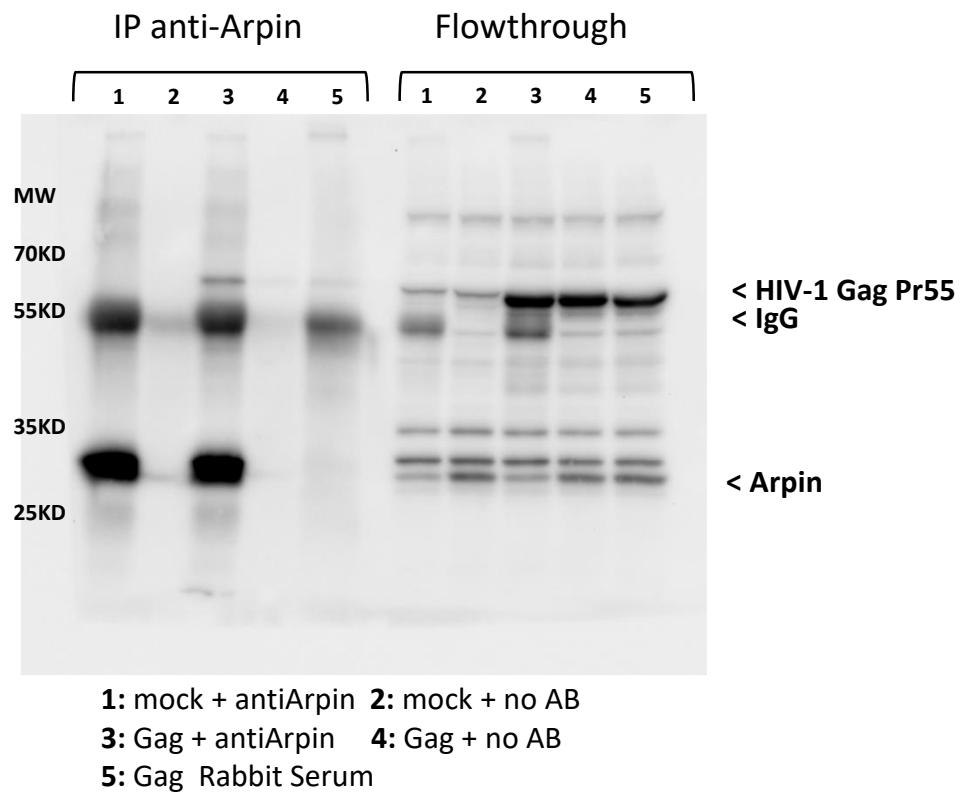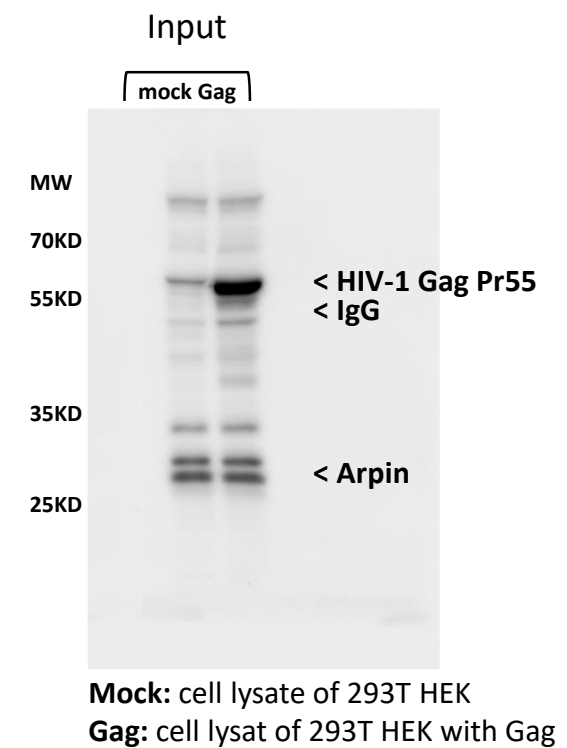

Supplement: Supplementary file 8 — Source Data [file 41467_2023_41940_MOESM8_ESM.zip › Source data western blots.pdf]
